# Evolutionary rate and gene expression across different brain regions

**DOI:** 10.1186/gb-2008-9-9-r142

**Published:** 2008-09-23

**Authors:** Tamir Tuller, Martin Kupiec, Eytan Ruppin

**Affiliations:** 1 School of Computer Sciences, Tel Aviv University, Ramat Aviv 69978, Israel; 2 Department of Molecular Microbiology and Biotechnology, Tel Aviv University, Ramat Aviv 69978, Israel; 3 School of Medicine, Tel Aviv University, Ramat Aviv 69978, Israel

## Abstract

**Background:**

The evolutionary rate of a protein is a basic measure of evolution at the molecular level. Previous studies have shown that genes expressed in the brain have significantly lower evolutionary rates than those expressed in somatic tissues.

**Results:**

We study the evolutionary rates of genes expressed in 21 different human brain regions. We find that genes highly expressed in the more recent cortical regions of the brain have lower evolutionary rates than genes highly expressed in subcortical regions. This may partially result from the observation that genes that are highly expressed in cortical regions tend to be highly expressed in subcortical regions, and thus their evolution faces a richer set of functional constraints. The frequency of mammal-specific and primate-specific genes is higher in the highly expressed gene sets of subcortical brain regions than in those of cortical brain regions. The basic inverse correlation between evolutionary rate and gene expression is significantly stronger in brain versus nonbrain tissues, and in cortical versus subcortical regions. Extending upon this cortical/subcortical trend, this inverse correlation is generally more marked for tissues that are located higher along the cranial vertical axis during development, giving rise to the possibility that these tissues are also more evolutionarily recent.

**Conclusions:**

We find that cortically expressed genes are more conserved than subcortical ones, and that gene expression levels exert stronger constraints on sequence evolution in cortical versus subcortical regions. Taken together, these findings suggest that cortically expressed genes are under stronger selective pressure than subcortically expressed genes.

## Abstract 2

Cortically expressed genes are more conserved than sub-cortical ones and gene expression levels exert stronger constraints on sequence evolution in cortical than in sub-cortical regions.

## Background

The evolutionary rate (ER) of a protein, the ratio between the rate of its nonsynonymous to the rate of its synonymous mutations, *dN/dS*, is a basic measure of evolution at the molecular level (for example, see [1,2]). (Throughout the report, when we talk about the ER of a gene we actually refer to the ER of its corresponding protein.) It is affected by many systemic factors, including gene dispensability, expression level, the number of protein interactions, and the recombination rate [3-7]. Notably, functionally related genes tend to have similar ERs [8,9]. The expression level of yeast genes has been observed to be markedly and negatively correlated with their ER [5,10], even when controlling for the dispensability of the genes [4]. This inverse relation extends to other eukaryotes (including humans and other vertebrates) [11]. Obviously, when considering the relationship between ER and gene expression in multicellular organisms, the expression levels of genes in different tissues and cell types should be considered separately. Indeed, previous studies [12-15] have shown that genes vary in their rates of evolution according to the tissues in which they are highly expressed, with genes expressed in the brain evolving at significantly slower rates than those expressed in other tissues. A general principle arising from such studies has been that tissue-specific genes have higher ERs than 'housekeeping' genes, which are broadly expressed in most tissues [16-18].

To explain this observation, the tissue-driven hypothesis of genomic evolution was recently proposed, starting from the probable assumption that genes influence phenotypic characters by their expression in specific tissues [19]. Accordingly, if a protein is expressed in several different tissues, then the evolution of its sequence may be under multi-tissue-specific constraints, resulting in a slower rate of evolution. Among genes with similar expression broadness (genes that are expressed in about the same number of tissues), those genes expressed in tissues that are presumably under more stringent evolutionary selection pressure (for example, neural tissues) generally tend to evolve more slowly than those that are expressed in tissues that presumably are under lesser selection pressure [19]. This hypothesis is concordant with the notion that each tissue is associated with a certain level of evolutionary constraints acting on the genes expressed in it, with the brain imposing more constraints than other tissues [15].

This study aims to go beyond previous investigations and to study the tissue-driven hypothesis at higher resolution, in an organ of central importance to human evolution: the brain. To this end, we examine the evolution of genes that are highly expressed in different brain tissues. Our work stems from the basic observation that the transcriptomes of different brain regions differ substantially from each other [20]. These differences are likely to be functionally significant, because they mainly involve genes that are associated with central functions such as signal transduction and neurogenesis [20].

First, we are interested in examining whether the basic inverse relationship between a gene's tissue specificity and its ER also holds in different brain regions. Second, we examine the ER of highly expressed genes in the more phylogenetically recent cortical brain regions, compared with the ERs of genes that are highly expressed in older brain regions. It was previously found that older genes (that arose earlier in evolution) tend to evolve more slowly than newer ones [21,22]. Does this finding translate to the brain tissue/region level? (Specifically, do genes expressed in older brain regions evolve more slowly than those expressed in new ones?) Third, we examine the extent to which the basic correlation between expression level and sequence conservation varies across brain regions, and learn from its variation about the selection forces that drive sequence evolution of highly expressed genes.

## Results

### Brain region-specific indices of gene expression and conservation

We analyze a dataset encompassing the expressions of 10,594 human genes, across 78 tissues (Additional data file 1) [23]. Twenty-one of these tissues are from different brain regions (Table 1). First, these brain regions can be broadly divided into two major phylogenetic classes: cortical regions, which are primarily characteristic of the mammalian lineage; and subcortical brain regions, which have a broad phyletic distribution [24]. (No other vertebrates have a structure that clearly resembles the isocortical regions studied here [25].) Second, the brain regions are divided into four major developmental classes, including those that develop from the embryonic forebrain, midbrain, hindbrain, and spinal cord [26]. For each brain region we define a gene set, composed of the genes that are over-expressed in that particular region. A gene is defined as over-expressed in a given brain region if its expression is at least 2 standard deviations higher than the mean of its expression across all of the regions.

**Table 1:** The 21 brain regions examined in this study and their characteristics

Index	Brain tissue	Developmental origin	Median ER (human lineage)	Median ER (mouse-human)	Frequency of mammalian genes	Frequency of mammalian genes for SCS	Frequency of primate genes	Brain region specificity index (T_max_)	Correlation (*P *value) between ER and expression level (human-mouse)
1	Dorsal root ganglion	Spinal cord	0.31	0.167	0.17	0.12	0.016248	0.12	-0.0747 (1.4 × 10^-14^)
2	Medulla oblongata	Hindbrain	0.28	0.102	0.12	0.14	0.005525	0.1	-0.1844 (<10^-16^)
3	Pons	Hindbrain	0.27	0.13	0.17	0.13	0.013158	0.11	-0.1442 (<10^-16^)
4	Spinal cord	Spinal cord	0.25	0.127	0.13	0.16	0.003704	0.11	-0.1664 (<10^-16^)
5	Olfactory bulb	Subpallium (forebrain)	0.28	0.126	0.09	0.08	0	0.14	-0.1475 (<10^-16^)
6	Trigeminal ganglion	Different developmental origin	0.31	0.161	0.17	0.17	0.021223	0.12	-0.0445 (4.7 × 10^-6^)
7	Ciliary ganglion	Different developmental origin	0.29	0.154	0.14	0.12	0.015152	0.12	-0.0710 (2.6 × 10^-13^)
8	Superior cervical ganglion	Different developmental origin	0.31	0.161	0.16	0.13	0.013664	0.12	-0.0335 (5.7 × 10^-4^)
9	Cerebellum	Hindbrain	0.14	0.083	0.08	0.09	0.004357	0.11	-0.1799 (<10^-16^)
10	Cerebellum peduncles	Hindbrain	0.17	0.096	0.08	0.09	0.005102	0.12	-0.1846 (<10^-16^)
11	Hypothalamus	Diencephalon (forebrain)	0.27	0.12	0.08	0.08	0	0.11	-0.1922 (<10^-16^)
12	Thalamus	Diencephalon (forebrain)	0.19	0.103	0.1	0.04	0	0.10	-0.1903 (<10^-16^)
13	Subthalamic nucleus	Diencephalon (forebrain)	0.23	0.11	0.14	0.2	0.005362	0.10	-0.1811 (<10^-16^)
14	Caudate nucleus	Subpallium (forebrain)	0.26	0.105	0.09	0	0	0.11	-0.1817 (<10^-16^)
15	Globus pallidus	Subpallium (forebrain)	0.23	0.107	0.12	0.1	0.010753	0.1	-0.1733 (<10^-16^)
16	Amygdala	Subpallium (forebrain)	0.21	0.084	0.09	0.11	0.002151	0.1	-0.2294 (<10^-16^)
17	Cingulate cortex	Pallium (forebrain)	0.2	0.094	0.07	0.05	0	0.1	-0.1931 (<10^-16^)
18	Occipital lobe	(pallium) (forebrain)	0.2	0.089	0.05	0	0	0.09	-0.2199 (<10^-16^)
19	Parietal lobe	Pallium (forebrain)	0.22	0.119	0.1	0.14	0	0.1	-0.1893 (<10^-16^)
20	Temporal lobe	Pallium (forebrain)	0.21	0.104	0.13	0.1	0.006897	0.11	-0.2214 (<10^-16^)
21	Prefrontal cortex	Pallium (forebrain)	0.22	0.089	0.08	0.06	0.002299	0.1	-0.1747 (<10^-16^)

ER, evolutionary rate; SCS, specific characteristic set.

Our dataset encompasses 4,919 genes that are over-expressed in at least one brain region. When this list of genes is analyzed using the Gene Ontology (GO) process category, enrichment for neural functions is found, attesting to their biologic relevance (Additional data files 2 and 3). We focus on over-expressed genes, following previous studies of expression signatures of different brain regions [27]. Notably, the enriched GO categories of under-expressed genes do not include neurally related categories (Additional data file 4). We additionally define for each brain region a more stringent specific characteristic set (SCS), which includes genes that are solely highly expressed in this region and in no other region.

We denote the brain expression specificity T_max _to be the ratio between the highest expression level of a gene in a brain region and the sum of its expression levels across all 21 brain regions. The coefficient of variance (CV) of a gene is the variance of its expression levels across brain regions divided by its mean expression. The CV thus estimates the expression variability of each gene across regions. The ERs of all of the genes along the human lineage and along a longer, mammalian range (human-mouse) were computed (see Materials and methods, below) and were used to extract the median ERs of over-expressed genes in each brain region (columns 4 and 5 in Table 1). Because the development of cortical and subcortical regions is not a human-specific morphologic trait but already a mammalian one, we primarily report the results in the main text using the human-mouse lineage for estimating ERs, and provide the corresponding (qualitatively similar) results using the mammalian ERs in the supplementary materials (Additional data file 5 [Supplementary note 1]).

A four-level estimation of gene age was computed following a procedure similar to that in [21,22], by searching for homologs of each human gene in four sets of organisms (mammals, fish, insects and worms, and yeast and plants). A gene with a homolog only in mammals is considered a mammalian gene, and a gene with a homolog only in the primates is considered a primate gene (see Materials and methods, below). Table 1 depicts the age group, developmental origin, median ER (human lineage), median ER (human-mouse), frequency of mammalian genes, frequency of mammalian SCS genes, frequency of primate genes, and mean T_max _for each brain region, computed for the genes over-expressed in each region. The last column in the table includes the correlation between the genes' ER (human-mouse), and their expression levels in each region and a corresponding *P *value. It can be seen that there is excellent agreement between the maximally expressed gene set and the more stringent SCS gene set, both in their ER (with both the mammalian and human estimators) and in gene age.

### Evolutionary rate, gene age and gene expression in cortical *vs *sub-cortical regions

**Figure 1:**
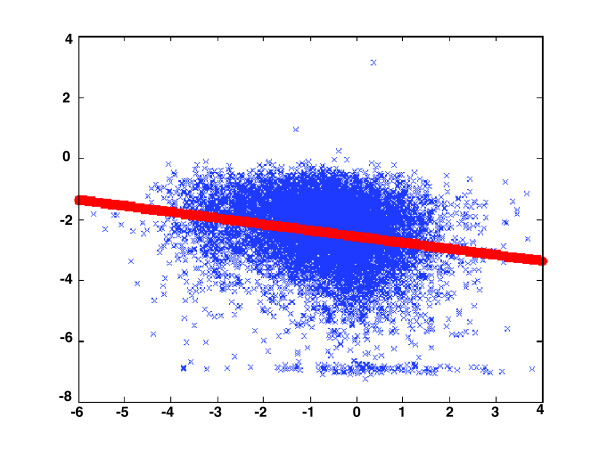
Expression in the prefrontal cortex versus ER. Expression in the prefrontal cortex is presented on the x-axis (log scale) and ER (human-mouse) on the y-axis (log scale). ER, evolutionary rate.

**Figure 2:**
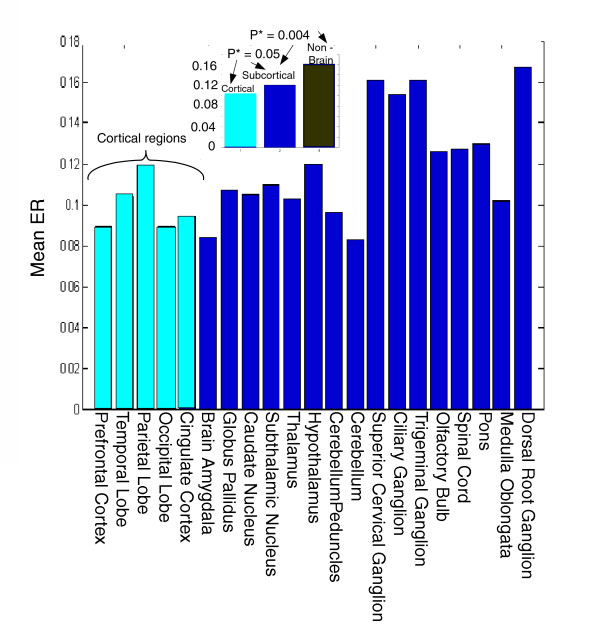
Median ER in each brain region. Presented in the top-left corner is a comparison of the (aggregated) medians of ER in cortical brain regions, subcortical brain regions, and somatic, nonbrain tissues. ER, evolutionary rate.

We computed the correlation between the ER and gene expression levels in each region, and the median ER of its over-expressed genes (see Figures 1 and 2 for the ER of each tissue separately, and gene expression in the prefrontal cortex versus their ERs).

The ER of genes that are highly expressed in cortical brain regions is significantly lower than that of genes highly expressed in noncortical brain regions (mean ER of 0.1016 versus 0.1378; *P *< 10^-16^). The medians of the SCS genes of the regions in the two sets exhibit a similar trend (*P *= 6 × 10^-5^).

This finding remains robust also after controlling for the total gene expression of genes and their expression breadth (Additional data file 5 [Supplementary note 2]). Similarly, gene compactness and gene essentiality, which are additional important determinants of mammalian protein ER (essential genes have lower ERs, and compact genes have higher ER values [28]), cannot explain the difference in ERs between cortical and subcortical genes. The frequency of essential genes among cortical genes is lower (15%) than among subcortical genes (16%), ruling out the possibility that the lower ER of cortical genes is due to the fact that they include greater numbers of lethal genes; and the fact that the ER of cortical genes is significantly lower than that of noncortical genes remains robust even after controlling for gene compactness (Additional data file 5 [Supplementary note 3]).

Finally, genes that are over-expressed in both parts of the brain have significantly lower median ERs than genes that are over-expressed in nonbrain regions (Figure 2).

**Figure 3:**
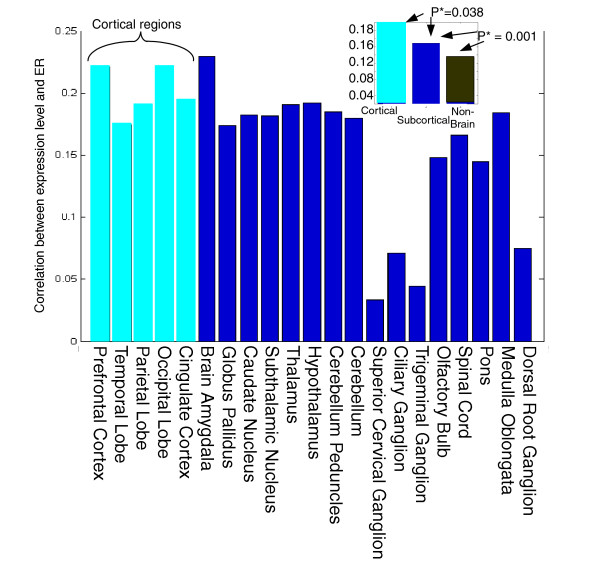
Correlation between ER and the expression levels in each brain region. Presented in the top-right corner is the mean correlation between ER and expression in cortical brain regions, subcortical brain regions, and somatic, nonbrain tissues. ER, evolutionary rate.

The correlation between the ER and expression levels is higher in cortical than in subcortical brain regions (*P *= 0.038; Figures 1 and 3), and higher in brain tissues than in other tissues. The raw mean expression levels in cortical regions is slightly lower than the subcortical regions (514 versus 518), thus ruling out the possibility that this finding is actually an indirect consequence of lower expression levels in subcortical versus cortical regions (because, hypothetically, lower expression levels could transcribe to a decreased signal-to-noise ratio, and hence to decreased ER/expression correlations).

We repeated our analysis (ER and ER/expression correlations in cortical and subcortical regions) in two other organisms: *Mus musculus *(mouse) and *Pan troglodytes *(chimp). Although the gene expression measurements of these two organisms is less abundant than in human, in both cases the ER of the cortical genes was lower than that of the subcortical genes (Additional data file 5 [Supplementary notes 4 and 5] and Additional data files 6 and 7).

Interestingly, the prefrontal cortex exhibits the greatest correlation between gene expression and ER among all of the 78 tissues (see Additional data files 8 and 9 for the ERs in all of the tissues, and the correlation between ER and expression level in each tissue). This cortical region is known to be associated in primates and humans with complex associative cognitive tasks such as those involving delayed response and working memory. Other interesting phenomena are the very low ER of the cerebellum, and the very low correlation between expression level and ER in the various ganglia. We verified that even after removing these tissues the ER of cortical regions is still lower than that of subcortical regions (*P *= 1.3 × 10^-13 ^when considering all of the corresponding genes). The very low correlation between expression level and ER observed in the various ganglia may perhaps arise from the very small tissue volume of the latter, which may attenuate their effect in determining the ERs of their highly expressed genes. The high conservation of genes that are highly expressed in the cerebellum is a quandary; it is not a result of potential lower tissue specificity of these genes, which is not statistically different from the tissue specificity of the subcortical or all brain genes. However, the cerebellar genes have higher mean expression levels than the subcortical genes (1.09 versus 0.72; *P *< 10^-16^), which can partially explain their higher conservation.

The frequency of mammal-specific genes is higher in the highly expressed gene sets of subcortical brain regions than in those of cortical regions (with mean frequencies of 0.121 versus 0.086; *P *= 0.03). This difference remains similar also when considering primate-specific genes. The mean frequency is 0.0073 in subcortical versus 0.0018 in cortical regions (*P *= 0.05). This difference is surprising, but it is consistent with the negative correlation found between the genes' age and ER (-0.23; *P *< 10^-16^). A similar inverse correlation across all tissues was previously observed [21,22]. Genes that are expressed in cortical regions have higher mean expression levels across brain tissues than genes expressed in subcortical regions (1.6 versus 1; *P *= 2.4 × 10^-10^) and across all somatic tissues (0.9 versus 0.7; *P *< 10^-16^). This fact can partially explain the lower ER values of cortical genes, because these genes are likely to be subjected to diverse simultaneous selective pressures.

### Relation between ER, expression level, and region specificity

Previous work has demonstrated that housekeeping genes tend to evolve more slowly than tissue-specific genes [17]. Gene expression across brain tissues manifests a similar region-specificity relation between gene expression and ER; genes highly expressed in fewer brain regions have higher ER values (the Spearman correlation between T_max _and ER along the mammalian lineage is 0.131 [*P *< 10^-16^] and along the human lineage it is 0.0504 [*P *< 2 × 10^-7^). A similar trend is observed by noting that genes with higher CV levels have higher ER values (Spearman correlation along the mammalian lineage is 0.1269 [*P *< 10^-16^] and along the human lineage it is 0.0447 [*P *= 4.1 × 10^-6^]).

**Figure 4:**
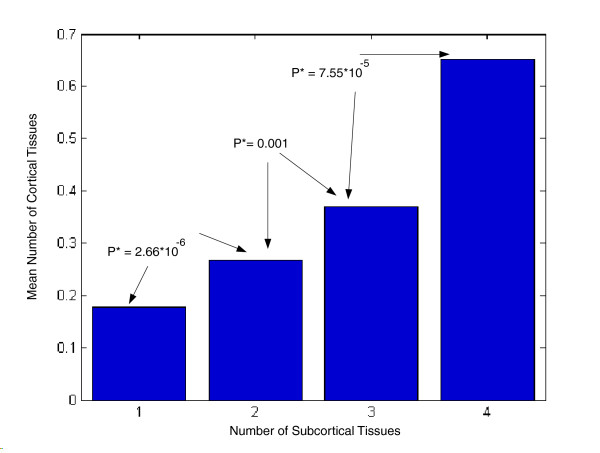
Expression in the cortical regions *vs. *expression in the subcortical regions. Shown is the mean number of cortical brain regions in which a gene is highly expressed (presented on the y-axis), given the number of subcortical brain regions in which the same gene is highly expressed (x-axis). Genes that are highly expressed in more subcortical regions tend to be highly expressed in more cortical regions.

Genes that are expressed in cortical regions are also less region-specific than those expressed in subcortical regions; the T_max _values of genes expressed in each cortical region are significantly higher than those expressed in each subcortical regions (mean T_max _of 0.10 versus 0.12; *P *= 0.02). Aggregating all genes expressed in cortical or subcortical regions together yields a mean T_max _of 0.106 for cortical regions and mean T_max _of 0.116 for subcortical ones (*P *= 3.4 × 10^-12^), showing a similar trend. Thus, genes that are highly expressed in cortical regions have a higher expression breadth that may partly (Additional data file 5 [Supplementary note 6]) account for their overall lower ER values. This reduced region specificity of cortical genes may arise due to a 'preferential attachment'-like process [29], in which the genes that are highly expressed in the more recent cortical regions in the mammalian lineage tend to be those that already have a broad expression breadth in subcortical regions. In accordance with that, we find a marked correlation of 0.28 (*P *< 10^-16^) between the number of cortical and subcortical regions in which a gene is expressed (Figure 4).

### ER and gene expression: a developmental perspective

We divided the brain into five main developmental areas [26]: three forebrain areas, including the pallium, subpallium, and the diencephalon; the hind brain; and the spinal cord (our data do not include midbrain structures, and additionally includes three cranial nuclei of different developmental origins).

**Figure 5:**
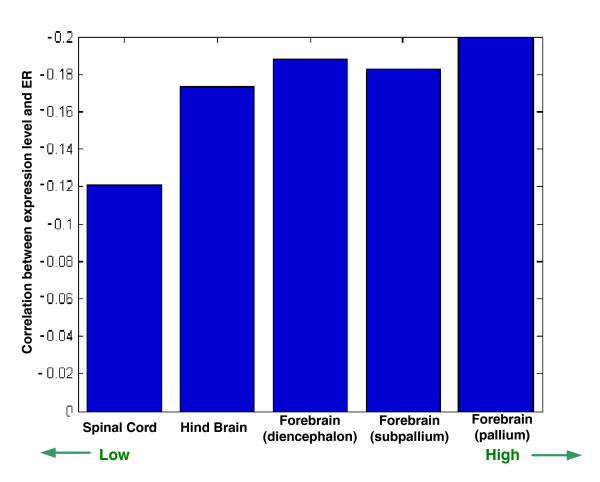
Correlation of expression levels in different embryonic developmental origins with ER. Shown is the mean correlation of expression levels with ER (human-mouse), for regions belonging to five different embryonic developmental origins. The latter are ordered on the x-axis in accordance with their height on the cranial vertical axis during early embryonic stages (spinal cord is the lowest, and Forebrain [pallium] is the highest). As evident, these ER/expression correlations are ordered by their cranial vertical location (Spearman rank correlation of 0.9; *P *= 0.037). A similar result was observed when computing the ER using the human lineage (human-chimpanzee; Additional data file 10). ER, evolutionary rate.

These five developmental areas have an ordered placement along the cranial vertical axis, with the spinal cord being the lowest [26,30], then the hindbrain, followed by the diencephalon, the subpallium, and the pallium (the highest). The correlation between ER and gene expression levels exhibits an interesting pattern; their magnitude manifests a significant correlation with the region location on the cranial vertical axis (Spearman ranked correlation of 0.9 [*P *= 0.037]when averaging the regions of each developmental area, and Spearman ranked correlation of 0.5 [*P *= 0.034] when considering each region separately; Figure 5, and Additional data files 10 and 11). This finding reinforces the observations made in the previous sections, suggesting that the genes' ERs are under tighter influence of their expression levels in cortical regions (which are of pallial origin).

## Discussion

Previous studies have shown that the rate of evolution among brain-expressed genes is probably lower (or at most equal) in humans compared with chimpanzee and old world monkeys (for instance, most recently in [31]). Slower sequence evolution of tissue/region-specific genes is a probable indicator of stronger selective constraints operating on the region in hand. Hence, the overall sequence conservation of highly expressed brain genes makes them an interesting subject for the further study of the basic relation between gene expression and ER. To this end, we find that cortically expressed genes are more conserved than subcortical ones, and that gene expression levels exert stronger constraints on sequence evolution in cortical versus subcortical regions. Taken together, these findings support the view that cortically expressed genes are under stronger selective pressure than subcortically expressed genes.

One possible mechanism that can partially explain these findings is the overall broader tissue distribution of cortically expressed genes, but other nonexclusive mechanisms may take part. For instance, it is possible that there are more frequent genetic and protein interactions among highly expressed genes in the cortical regions, which are known to be correlated with reduced ER levels [7]. The cellular complexity (types of cells and their distribution) of the regions studied is different, which may further determine different and complex evolutionary constraints in each region. Another factor potentially influencing these regional differences is the sex bias of genes, because it has been suggested that the expression of genes that are more pleiotropic (or, in terms of our work, that have a greater tissue expression breadth) is less sex biased [32], and that sex-dependent allelic effects cannot maintain polygenic variation [33]. Thus, the exact mechanisms underlying our findings are probably subject to quite complex interplay that remains to be further explored.

The magnitudes of some of the ER/expression correlations reported here are lower than in yeast (see Figure 1 for gene expression in the prefrontal cortex versus their ERs), even though these correlations are highly significant. In the case of the yeast (for example [5]), the respective correlation found is around 1.5 times higher. There are three main reasons that may explain this difference. First and foremost, in contrast to the yeast, humans are multicellular organisms with hundreds of distinct cell types and diverse tissues; thus, gene ER in humans is likely to be under a large variety of (sometimes perhaps counteracting) selection forces, resulting in a lower correlation with gene expression in any single specific cell/tissue type [28]. Second, because this study focuses on human brain regions, we have estimated ER values along shorter evolutionary time periods (the past 6.5 to 10 million years of the human lineage, after the human-chimp split [34,35], and the 50 to 100 million years corresponding to the human-mouse split), which is in contrast to the much longer time spans employed for estimating ER in the yeast studies. Indeed, when using ER estimates using the human-mouse lineage, we obtain ER/expression correlations that are two times higher than those obtained when using ER estimates from the shorter period, human-chimp lineage. Third, the sets of genes studied differ markedly, with the number of genes included in this study being two to three times higher than the number of genes examined in previous yeast studies (larger datasets usually increase the correlations but may decrease their significance).

Cortical regions, at least in their extensive mammalian form, are more recent than subcortical regions, which have a broader phyletic distribution. The ER of cortically expressed genes is yet slower than that of subcortically expressed genes. This is in contrast to the findings at the gene level, at which the ER of younger genes is higher than that of older ones [21]. This appears paradoxical at first, because one would perhaps expect that genes that are highly expressed in the more recently evolving cortical brain regions would be younger than the genes that are highly expressed in subcortical regions. However, this is not the case; highly expressed cortical genes tend also to be highly expressed in many subcortical regions, and thus both types of regions are composed of both younger and older highly expressed genes (with cortical areas being actually composed of older genes than subcortical regions, on average). Furthermore, although we find that cortically expressed genes are more conserved than subcortical ones, this does not necessarily imply that cortical regions offer more stringent 'environments' for gene evolution than subcortical regions, because this excess conservation may arise from their broader, somatic tissue distribution. However, the tighter correlation between ER and expression levels that characterizes cortically expressed genes does point to the fact that the cortex may form a more stringent environment for gene evolution than other brain and somatic tissues, as one may intuitively expect [19]. (Obviously, in turn, it is also possible that the rates of gene evolution may play an important role in shaping their expression profiles in the cortex.)

There are many definitions for the tissue/region specificity of genes (for example, based on expressed sequence tags data, serial analysis of gene expression data, literature [36,37], or gene expression [as was adopted here]). Each of the definition may give rather different sets of genes. Currently, there are no available datasets based on expressed sequence tags, serial analysis of gene expression, or literature that provide information about brain regional specificity. Hence, we have focused on the gene expression definition of region specificity. A comparison of our results with those based on other tissue specificity definitions will have to be deferred until the corresponding biologic information becomes available.

Finally, the results reported in Figure 5 are intriguing, generalizing in a way the results reported in Figures 1 to 3. Whereas Figures 1 to 3 report that cortical regions exhibit a correlation between ERs and tissue gene expression levels in cortical versus subcortical regions, Figure 5 shows that this correlation tends to be stronger for vertically higher regions in the developmental axis. each point in Figure 5 corresponds to the correlation between ER and expression levels of 10,594 genes in all of the regions in a developmental area, the reported correlation values are highly robust. Thus, drawing an analogy from the observation that cortical regions are evolutionary more recent than subcortical ones [24], one may (perhaps boldly) speculate that regions located higher on the vertical axis at brain development are also more evolutionarily recent. However, because even the basic claim that cortical regions are more recent is not accepted by everyone, care should obviously be taken with formulating such hypotheses. Their examination should await the accumulation of additional gene expression samples from more brain tissues and from more mammalian species.

## Conclusion

Our findings support the view that cortically expressed genes are under stronger selective pressure than subcortically expressed genes. They may also suggest that regions that are located higher on the vertical axis at brain development are also more evolutionarily recent. These findings should be re-examined when additional biological data (for example, gene expression samples from more brain tissues and from more mammalian species) will become available.

## Materials and methods

### Computation of ERs

We used two estimations of human ERs: long-term ERs along the mammalian lineage (human-mouse *dN*/*dS*) that were downloaded from European Bioinformatics Institute (EBI) BioMart (BioMart July 2008), and short-term ERs along the human lineage (human-chimpanzee *dN*/*dS*), whose computations are described in the following subsection.

### Computing gene ERs along the human and chimp lineages

We downloaded the orthologous groups of *Homo sapiens *(humans), *Pan troglodytes *(chimp), and *Macaca mulatta *(macaque) from EBI BioMart Homology (BioMart November 2007). We considered only sets that include orthologs in all three species. Sets of homologs that did not include exactly one representative in each organism were removed from our dataset, in order to filter out paralogs and to avoid potential errors in ER estimation caused by duplication events (today, there are no reported cases of horizontal gene transfer) between *Primates*; see, for instance, [38]). This procedure resulted in a total of 15,176 orthologous gene sets.

In the next step, stop codons were removed from each gene and the genes were translated to sequences of amino acids. The corresponding amino acid sequences of each orthologous gene set were aligned by CLUSTALW 1.83 [39], with default parameters. By using amino acids as templates for the nucleotide sequences and by ignoring gaps, we generated gap-free multiple alignments of the three orthologous proteins in each orthologous set and their corresponding coding sequences.

**Figure 6:**
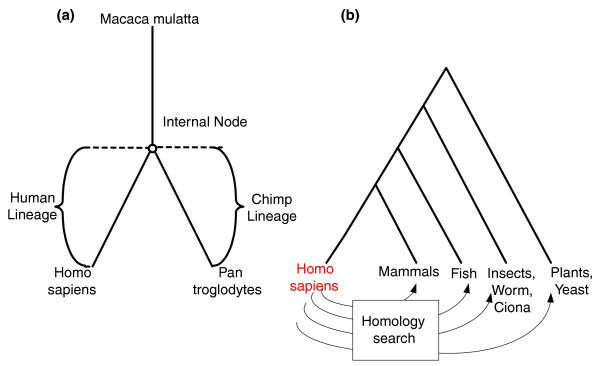
Illustration of the procedures that were used for estimating ER and gene age. **(a) **Phylogeny of the three primates whose genes were used to compute the ERs along the human lineage. **(b) **Illustration of the procedure and the phylogenetic tree used to estimate gene age. ER, evolutionary rate.

Given the alignments of each set of orthologs and given the phylogenetic tree of the three primates (Figure 6a), we used the codeml program in PAML for the joint reconstruction of ancestral codons in the internal nodes of the phylogenetic tree [40] (the ancestor of the human and chimp; Figure 6a). This reconstruction induced the sequence of the ancestral proteins and their corresponding ancestral DNA coding sequences. We hence obtained sets of four sequences: three from the previous step (corresponding to the three leaves of the phylogenetic tree) plus one reconstructed sequences of the internal node of the phylogenetic tree. We denote such a set of four sequences a 'complete ortholgous set'. For each complete ortholgous set, we computed the *dN *(the rate of nonsynonymous substitutions) and *dS *(the rate of synonymous substitutions) along the linage to the human (the branch between the internal node and the human node; see Figure 6a) by the y00 program in PAML [41,42]. The ER of a gene is the *dN *divided by the *dS *of its corresponding complete ortholgous set along the human lineage (the *dN*/*dS *along the human lineage).

Similarly, we computed that *dN *and *dS *and a corresponding ER (*dN*/*dS*) along the chimp linage.

### Computing ERs of tissues

The ER of a tissue/region is the median ER of all the genes that are over-expressed in that tissue (genes that are 2 standard deviations higher than their mean expression across all of the tissues). We used median instead of average because the analyzed set of genes included genes with *dS *= 0 (ER equals infinity).

It is important to note that all of the results reported here are remarkably robust to changing the cutoff of 2 standard deviations. For example if we choose a cutoff of 3 standard deviations, then the average median ER of cortical brain regions is 0.1532 versus 0.2387 in subcortical brain regions, and 0.2914 in nonbrain tissues (*P *= 0.014 and *P *= 0.002 respectively).

### Gene expression data

The gene expression of 78 tissues (including 21 brain regions) was downloaded from the work of [23]. All of the analyzed gene expression measurements were from the same technology (Affymetrix GeneChip Human Genome U133 Array Set HG-U133A; Affymetrix Inc., Santa Clara, CA, USA) and included two technical repeats (that we averaged). A list of all of the tissue names and other properties appears in Additional data file 12. The human tissue samples were obtained from several sources: Clinomics Biosciences (Pittsfield, MA, USA), Clontech (Palo Alto, CA, USA), AllCells (Berkeley, CA, USA), Clonetics/BioWhittaker (Walkersville, MD, USA), AMS Biotechnology (Abingdon, Oxfordshire, UK), and the University of California at San Diego. When samples from four or more subjects were available, equal numbers of male and female subjects were used to make two independent pools; when fewer than four samples were available, RNA samples were pooled, and duplicate amplifications were performed for each pool. (More details appear in supporting Table 1 in [23].) We averaged the signals of all of the probes of each gene to obtain a final set of 10,594 genes with both ER measurements and gene expression measurements across all tissues.

Gene expression of *M. musculus *(mouse) was downloaded from [43]. It includes gene expression from 61 tissues. Fourteen of these tissues are brain tissues (2 cortical and 12 subcortical). The mouse gene expression appears in Additional data file 13. The gene expression of *P. troglodytes *(chimp) was downloaded from the Gene Expression Omnibus database [48] (GDS2678 record). It included 12 brain tissues; one of them was subcortical and the others were all cortical.

### Estimating gene age

An estimation of gene age was obtained following the procedure described in [21,22] (Figure 6b). First, we used the homology search engine of BioMart (November 2007) to find all of the orthologs of each of the human genes in a set of 34 organisms (the list of organisms appear in Additional data file 14, and phyletic patterns of all the human genes appear in Additional data file 15). Next, the organism set was divided into four groups: group 1, mammals (youngest group); group 2, fish; group 3, insects, worm, ciona; and group 4, plants and yeast (oldest group). The age of each of the human genes was determined according to the oldest organism group with a homolog of the gene (Figure 6b). Accordingly, a gene that has homolog(s) only in group 1 is named a mammalian gene, whereas a gene with homolog(s) in group 4 is very old. We also performed an additional study of primate specific genes, defined as human genes that have homolog(s) only in the primates (*P. troglodytes *or *M. mulatta*).

### GO enrichments

Hypergeometric functional GO enrichment of an over-expressed set of genes, including a correction for multiple testing, was computed by the FuncAssociate [49].

### Gene length and gene essentiality

Information about gene and protein lengths was downloaded from EBI BioMart (BioMart August 2008). The information about gene essentiality was based on the mouse phenotypic data, and was downloaded from Mouse Genome Informatics database [50]. Human genes whose mouse orthologs have knockout phenotype of lethality or sterility were defined as essential. That is, those entries possessing embryonic lethality (MP: 0002080), prenatal lethality (MP: 0002081), survival postnatal lethality (MP: 0002082), premature death or induced morbidity (MP: 0002083), reproductive system phenotype (MP: 0002161, MP:0005389), lethality postnatal (MP:0005373), or lethality prenatal/perinatal (MP:0005374).

## Abbreviations

CV: coefficient of variance; *dN*: rate of nonsynonymous substitutions; *dS*: rate of synonymous substitutions; EBI: European Bioinformatics Institute; ER: evolutionary rate; GO: Gene Ontology; SCS: specific characteristic set.

## Authors' contributions

TT carried out all of the analysis. All authors participated in the design of the study. All authors were involved in drafting and writing the manuscript. All authors read and approved the final manuscript.

## Additional data files

The following additional data are available with the online version of this paper. Additional data file 1 is a table listing various parameters of the analyzed genes. Additional data file 2 is a table listing the GO enrichments for the genes that are over-expressed in the cortical regions. Additional data file 3 is a table listing the GO enrichments for the genes that are over-expressed in the subcortical regions. Additional data file 4 is a table listing the GO enrichment categories for the genes that are under expressed in the cortical and in the subcortical brain regions. Additional data file 5 includes the Supplementary notes (Supplementary notes 1 to 6). Additional data file 6 is a table that includes the ER/expression correlation and ER in each mouse tissue. Additional data file 7 is a table that includes the ER/expression correlation in the chimp brain tissues. Additional data file 8 is a figure that depicts the following: (A) median ER (human lineage) in brain tissues and other tissues; (B) median ER in each brain region; (C) the correlation between ER (human lineage) and expression level in each tissue; and (D) the correlation between ER and the expression levels in each brain region. Additional data file 9 is a figure that depicts the following: (A) median ER (human-mouse *dN*/*dS*) in brain tissues and other tissues; and (B) the correlation between ER (human-mouse *dN*/*dS*) and expression level in each tissue. Additional data file 10 is a figure that depicts the mean correlation of expression levels with ER (human lineage), for regions belonging to five different embryonic developmental origins. Additional data file 11 is a figure that depicts the mean ER (mouse-human) for regions belonging to five different embryonic developmental origins. Additional data file 12 is a table listing various properties of the analyzed tissues. Additional data file 13 is a table that includes the mouse gene expression and ER. Additional data file 14 is a table with the set of organisms that was used for estimating gene ages. Additional data file 15 is a table that includes the phyletic patterns of all the human genes.

## Supplementary Material

Additional data file 1 Presented is a table listing various parameters of the analyzed genes. Click here for file

Additional data file 2 Presented is a table listing the GO enrichments for the genes that are over-expressed in the cortical regions. Click here for file

Additional data file 3 Presented is a table listing the GO enrichments for the genes that are over-expressed in the subcortical regions. Click here for file

Additional data file 4 Presented is a table listing the GO enrichment categories for the genes that are under expressed in the cortical and in the subcortical brain regions. Click here for file

Additional data file 5 Presented are the Supplementary notes (Supplementary notes 1 to 6). Click here for file

Additional data file 6 Presented is a table that includes the ER/expression correlation and ER in each mouse tissue. Click here for file

Additional data file 7 Presented is a table that includes the ER/expression correlation in the chimp brain tissues. Click here for file

Additional data file 8 Presented is a figure that depicts the following: (A) median ER (human lineage) in brain tissues and other tissues; (B) median ER in each brain region; (C) the correlation between ER (human lineage) and expression level in each tissue; and (D) the correlation between ER and the expression levels in each brain region. Click here for file

Additional data file 9 Presented is a figure that depicts the following: (A) median ER (human-mouse *dN/dS*) in brain tissues and other tissues; and (B) the correlation between ER (human-mouse *dN/dS*) and expression level in each tissue. Click here for file

Additional data file 10 Presented is a figure that depicts the mean correlation of expression levels with ER (human lineage), for regions belonging to five different embryonic developmental origins. Click here for file

Additional data file 11 Presented is a figure that depicts the mean ER (mouse-human) for regions belonging to five different embryonic developmental origins. Click here for file

Additional data file 12 Presented is a table listing various properties of the analyzed tissues. Click here for file

Additional data file 13 Presented is a table that includes the mouse gene expression and ER. Click here for file

Additional data file 14 Presented is a table with the set of organisms that was used for estimating gene ages. Click here for file

Additional data file 15 Presented is a table that includes the phyletic patterns of all the human genes. Click here for file
