# Supplementary material for: Evolutionary rate and gene expression across different brain regions
Source: Genome Biol. 2008 Sep 23;9(9):R142. doi: 10.1186/gb-2008-9-9-r142 (PMC2592720; doi:10.1186/gb-2008-9-9-r142)
Supplement: Additional data file 5 — Presented are the Supplementary notes (Supplementary notes 1 to 6). [file gb-2008-9-9-r142-S5.doc]

**Supplementary Notes for the work:**

**"Evolutionary Rate and Gene Expression Across Different Brain Regions"**

***Supplementary Note 1: The results reported in the paper are similar when using Evolutionary rates measured along the human lineage***

When considering the ERs along the human lineage we find, analogously to the findings with the mammalian lineage, that: (1) The ER of genes highly expressed in cortical brain regions is significantly lower than that of genes highly expressed in non-cortical brain regions (median ER of 0.21 vs 0.28, p-value < 2*10^-4^).

(2) The magnitude of the correlation between evolutionary rate and gene expression levels manifests a significant correlation with the region’s location on the cranial vertical axis (Spearman ranked correlation of 0.9, p-value = 0.037 when averaging the regions of each developmental area, and Spearman ranked correlation of 0.47 and p-value = 0.047 when considering each region separately).

***Supplementary Note 2: Evolutionary rate of cortical and sub-cortical regions after controlling for differences in gene expression levels and in expression breadth***

The mean expression levels (mean expression across all tissues) of the cortical genes are higher than those of non-cortical genes. To control for this potential confounding factor, we removed the cortical genes with top expression levels such that the mean expression of the remaining cortical genes and the sub-cortical genes is similar. Comparing the ER of these two groups, the ERs of the cortical genes were still significantly lower than those of sub-cortical genes (0.1015 vs 0.1378 in the cortical and sub cortical regions respectively, p-value < 10^-16^).

The expression breadth of a gene is the number of tissues where it is over expressed.. The mean expression breadth of the sub-cortical genes (3.77) is lower than that of cortical genes (4.25). To control for this potential confounding factor, we removed the cortical genes with top expression breadth such that the resulting mean expression breadth of cortical and sub-cortical genes will be identical. The ER of the cortical genes was still lower (0.1028 *vs* 0.1378 in the cortical and the sub cortical regions respectively, p-value = 6.46*10^-13^).

***Supplementary Note 3: Evolutionary rate of cortical and sub-cortical regions after controlling for gene compactness***

The average protein length of cortical genes is larger than the average protein length of sub-cortical genes (709.8 *vs.* 639.9). To check if this is the only cause for the difference in ER, we removed the cortical genes with top protein length such that the mean protein length of the remaining cortical genes and the sub-cortical genes is identical. The ER of the cortical genes was still lower (0.0966 vs 0.1363 in the cortical and the sub cortical regions respectively, p-value < 10^-16^).

When considering the length of the entire genes (including introns), the average gene length of cortical genes is larger than the average length of sub-cortical genes (123,190 *vs.* 93,964). To check if this is the only cause for the difference in ERs, we removed the cortical genes with top gene length such that the mean gene length of the remaining cortical genes and sub-cortical genes will be identical. The ER of the cortical genes was still lower (0.1032 vs 0.1378 in the cortical and the sub cortical regions respectively, p-value < 1.44*10^-14^).

***Supplementary Note 4: Evolutionary rate of cortical and sub-cortical regions in the mouse***

In mouse, as in human, the correlation between gene expression and ER is highest in cortical regions (due to the low number of mouse brain tissues, it was impossible to get a significant p-value when comparing the ER/expression correlation in the cortical and the sub-cortical tissues), and higher in the brain than in the non-brain tissues (p < 10^-16^, see Additional data file 6). The ER/expression correlation is significant across all the 61 mouse tissues (p-value < 10^-16^ for all the tissues).

When comparing the cortical and sub-cortical genes, the average ER of genes expressed in the cortical regions is lower (mean = 0.0907) than the ER of gene that are expressed in the other brain tissues (mean = 0.1060, p-value = 0.0055). When comparing all the brain and non-brain genes, the average ER in the brain tissues is lower than the ER in non-brain tissues (mean = 0.158, p-value < 10^-16^).

***Supplementary Note 5: Evolutionary rate of cortical and sub-cortical regions in the Chimpanzee***

In the Chimpanzee, the correlation with ER is lowest in the non-cortical tissue (the Cerebellum; see Additional data file 7). Due to the low number of Chimpanzee brain tissues, it was impossible to get a significant p-value when comparing the ER/expression correlation in the cortical and the sub-cortical tissues.

The ER/expression correlation is significant across all the 9 chimp tissues (p < 10^-16^ for all the tissues).

When comparing the cortical and sub-cortical genes, the average ER of genes expressed in the cortical regions (median = 0.2609) is lower than the ER of genes that are expressed in the non-cortical brain tissues (median = 0.2814, KS test, p-value = 0.05).

***Supplementary Note 6: Evolutionary rate of cortical and sub-cortical regions after controlling for Tmax***

The average Tmax of sub-cortical genes is larger than the average Tmax of cortical genes (0.116 *vs.* 0.106). To check if this is the only cause for the difference in ER, we removed the sub-cortical genes with top Tmax such that the mean Tmax of the remaining cortical genes and the sub-cortical genes is identical. The ER of the cortical genes was still lower (0.1016 vs 0.1324 in the cortical and the sub cortical regions respectively, p-value < 2.86*10^-12^).
