# Supplementary material for: Evolutionary rate and gene expression across different brain regions
Source: Genome Biol. 2008 Sep 23;9(9):R142. doi: 10.1186/gb-2008-9-9-r142 (PMC2592720; doi:10.1186/gb-2008-9-9-r142)
Supplement: Additional data file 8 — Presented is a figure that depicts the following: (A) median ER (human lineage) in brain tissues and other tissues; (B) median ER in each brain region; (C) the correlation between ER (human lineage) and expression level in each tissue; and (D) the correlation between ER and the expression levels in each brain region. [file gb-2008-9-9-r142-S8.doc]

**A.**

**B.**

**C.**

**D.**

**Supplementary Figure 1**. **A. Median ER (Human lineage) in brain tissues and other tissues. B. Median ER in each brain region. Top-left corner: Comparison of the (aggregated) medians of ER in cortical brain regions, sub-cortical brain regions, and somatic, non-brain tissues. C. The correlation between ER (Human lineage) and expression level in each tissue. D. The correlation between ER and the expression levels in each brain region. Top-right corner: The mean correlation between ER and expression in cortical brain regions, sub-cortical brain regions, and somatic, non-brain tissues.**
