# Supplementary material for: Evolutionary rate and gene expression across different brain regions
Source: Genome Biol. 2008 Sep 23;9(9):R142. doi: 10.1186/gb-2008-9-9-r142 (PMC2592720; doi:10.1186/gb-2008-9-9-r142)
Supplement: Additional data file 9 — Presented is a figure that depicts the following: (A) median ER (human-mouse dN/dS) in brain tissues and other tissues; and (B) the correlation between ER (human-mouse dN/dS) and expression level in each tissue. [file gb-2008-9-9-r142-S9.doc]

**A.**

**B.**

**Supplementary Figure 2**. **A. Median ER (Human-Mouse dN/dS) in brain tissues and other tissues. B. The correlation between ER (Human-Mouse dN/dS) and expression level in each tissue.**
