# Supplementary material for: Evolutionary rate and gene expression across different brain regions
Source: Genome Biol. 2008 Sep 23;9(9):R142. doi: 10.1186/gb-2008-9-9-r142 (PMC2592720; doi:10.1186/gb-2008-9-9-r142)
Supplement: Additional data file 10 — Presented is a figure that depicts the mean correlation of expression levels with ER (human lineage), for regions belonging to five different embryonic developmental origins. [file gb-2008-9-9-r142-S10.doc]

**Supplementary Figure 3.** **The mean correlation of expression levels with ER (Human lineage), for regions belonging to five different embryonic developmental origins. The latter are ordered on the x-axis in accordance with their height on the cranial vertical axis during early embryonic stages (Spinal Cord is the lowest, and Forebrain (pallium) is the highest). As evident, these ER/expression correlations are ordered by their cranial vertical location (Spearman rank correlation of 0.9, p-value = 0.037).**
