# Supplementary material for: Evolutionary rate and gene expression across different brain regions
Source: Genome Biol. 2008 Sep 23;9(9):R142. doi: 10.1186/gb-2008-9-9-r142 (PMC2592720; doi:10.1186/gb-2008-9-9-r142)
Supplement: Additional data file 11 — Presented is a figure that depicts the mean ER (mouse-human) for regions belonging to five different embryonic developmental origins. [file gb-2008-9-9-r142-S11.doc]

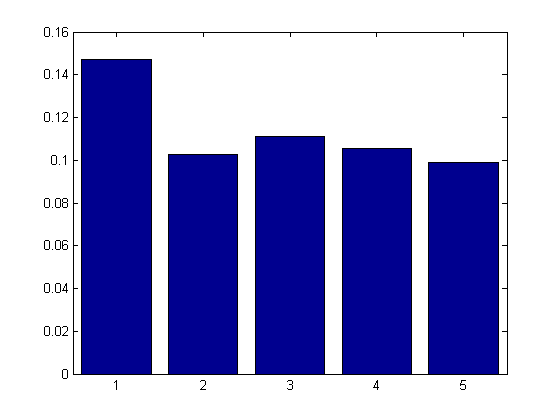


**Supplementary Figure 4. The mean ER (mouse human), for regions belonging to five different embryonic developmental origins. The latter are ordered on the x-axis in accordance with their height on the cranial vertical axis during early embryonic stages (Spinal Cord is the lowest, and Forebrain (pallium) is the highest). The ER, excluding the hind brain, are ordered by their cranial vertical location (Spearman rank correlation of 1, p-value = 0), with the hind brain the correlation is 0.7 (p-value = 0.1).**


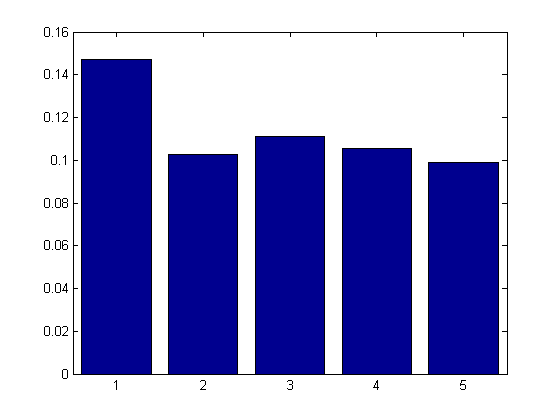


**Figure 4. The mean ER (mouse human), for regions belonging to five different embryonic developmental origins. The latter are ordered on the x-axis in accordance with their height on the cranial vertical axis during early embryonic stages (Spinal Cord is the lowest, and Forebrain (pallium) is the highest). The ER, excluding the hind brain, are ordered by their cranial vertical location (Spearman rank correlation of 1, p-value = 0).**

**Supplementary Figure 4x.** **The mean correlation of expression levels with ER (Human lineage), for regions belonging to five different embryonic developmental origins. The latter are ordered on the x-axis in accordance with their height on the cranial vertical axis during early embryonic stages (Spinal Cord is the lowest, and Forebrain (pallium) is the highest). As evident, these ER/expression correlations are ordered by their cranial vertical location (Spearman rank correlation of 0.9, p-value = 0.037).**
