# Supplementary material for: Evolutionary rate and gene expression across different brain regions
Source: Genome Biol. 2008 Sep 23;9(9):R142. doi: 10.1186/gb-2008-9-9-r142 (PMC2592720; doi:10.1186/gb-2008-9-9-r142)
Supplement: Additional data file 14 — Presented is a table with the set of organisms that was used for estimating gene ages. [file gb-2008-9-9-r142-S14.doc]

|  | **Group** | **Organism** | **Split time** | **References** |
| --- | --- | --- | --- | --- |
| 1 | -- | Homo Sapiens | ------- | ----- |
| 2 | 1. mammals | Pan troglodytes | 6.5 – 10MY | 35,44 |
| 3 | 1. mammals | Macaca mulatta | 23-35 MY | 35,44 |
| 4 | 1. mammals | Otolemur garnettii | 55-85 MY | 35,45 |
| 5 | 1. mammals | Tupaia belangeri | 55-95 MY | 351,45 |
| 6 | 1. mammals | Mus musculus | 55 - 113 MY | 35,34 |
| 7 | 1. mammals | Rattus norvegicus | 55 - 113 MY | 351,34 |
| 8 | 1. mammals | Cavia porcellus | 55 - 113 MY | 35,34 |
| 9 | 1. mammals | Spermophilus tridecemlineatus | 55 - 113 MY | ,3435 |
| 10 | 1. mammals | Oryctolagus cuniculus | 55 - 113 MY | 35,34 |
| 11 | 1. mammals | Loxodonta Africana | 81 - 193 MY | 35,34 |
| 12 | 1. mammals | Echinops telfairi | 81 – 193 MY | 35,34 |
| 13 | 1. mammals | Erinaceus europaeus | 81 – 193 MY | 35,34 |
| 14 | 1. mammals | Sorex araneus | 81 – 193 MY | 35,34 |
| 15 | 1. mammals | Bos taurus | 81 – 193 MY | 35,34 |
| 16 | 1. mammals | Canis familiaris | 81 – 193 MY | 35,34 |
| 17 | 1. mammals | Felis catus | 81 – 193 MY | 35,34 |
| 18 | 1. mammals | Myotis lucifugus | 81 – 193 MY | 35,34 |
| 19 | 2. Fish | Danio rerio | 378.3 – 416.9 MY | 35 |
| 20 | 2. Fish | Gasterosteus aculeatus | 378.3 – 416.9 MY | 35 |
| 21 | 2. Fish | Oryzias latipes | 378.3 – 416.9 MY | 35 |
| 22 | 2. Fish | Takifugu rubripes | 378.3 – 416.9 MY | 35 |
| 23 | 2. Fish | Tetraodon nigroviridis | 378.3 – 416.9 MY | 35 |
| 24 | 3. Insects, worm, Ciona. | Aedes aegypti | 531.5 - 1300 | 35,34 |
| 25 | 3. Insects, worm, Ciona. | Anopheles gambiaes | 531.5 - 1300 | 35,34 |
| 26 | 3. Insects, worm, Ciona. | Drosophila melanogaster | 531.5 - 1300 | 35,34 |
| 27 | 3. Insects, worm, Ciona. | Ciona intestinalis | 650 - 1500 | 4,463 |
| 28 | 3. Insects, worm, Ciona. | Ciona savignyi. | 650 - 1500 | 34,46 |
| 29 | 3. Insects, worm, Ciona. | Caenorhabditis elegans | 1100 - 1177 | 34 |
| 30 | 4. Plants, yeast. | Saccharomyces cerevisiae | 1100 - 1177 | 34,47 |
| 31 | 4. Plants, yeast. | Arabidopsis thaliana | 1300-1576 | 34,47 |
| 32 | 4. Plants, yeast. | Oryza sativa japonica | 1300 - 1576 | 34,47 |
| 33 | 4. Plants, yeast. | Oryza sativa indica | 1300 - 1576 | 34,47 |
| 34 | 4. Plants, yeast. | Zea mays | 1300 - 1576 | 34,47 |

**Supplementary Table 7.** The set of organisms that was used for estimating gene ages. The organisms are divided to four groups: 1) mammals (youngest), 2) Fish, 3) Insects, worm, Ciona , 4) Plants, yeast (oldest). The split time column includes the estimated split time (in millions of years) of the organism and human, the source of this information (cite) appear in the right column.
